# Hfe Actions in Kupffer Cells Are Dispensable for Hepatic and Systemic Iron Metabolism

**DOI:** 10.3390/ijms24108948

**Published:** 2023-05-18

**Authors:** Paul Knoop, Dilay Yilmaz, Rossana Paganoni, Peter Steele-Perkins, Andreas Gruber, Banu Akdogan, Hans Zischka, Kerstin Leopold, Maja Vujić Spasić

**Affiliations:** 1 Institute of Comparative Molecular Endocrinology, Ulm University, 89081 Ulm, Germany; paul.knoop@uni-ulm.de (P.K.);; 2 Institute of Analytical and Bioanalytical Chemistry, Ulm University, 89081 Ulm, Germany; 3 Institute of Molecular Toxicology and Pharmacology, Helmholtz Center Munich, 85764 Neuherberg, Germany; 4 Institute of Toxicology and Environmental Hygiene, Technical University Munich, School of Medicine, 80802 Munich, Germany

**Keywords:** HFE-hemochromatosis, Kupffer cells, macrophage, liver, iron, hepcidin

## Abstract

Mutations in the *HFE*/*Hfe* gene cause Hereditary Hemochromatosis (HH), a highly prevalent genetic disorder characterized by elevated iron deposition in multiple tissues. HFE acts in hepatocytes to control hepcidin expression, whereas HFE actions in myeloid cells are required for cell-autonomous and systemic iron regulation in aged mice. To address the role of HFE specifically in liver-resident macrophages, we generated mice with a selective *Hfe* deficiency in Kupffer cells (*Hfe*^Clec4fCre^). The analysis of the major iron parameters in this novel *Hfe^Clec4fCre^* mouse model led us to the conclusion that HFE actions in Kupffer cells are largely dispensable for cellular, hepatic and systemic iron homeostasis.

## 1. Introduction

The HFE is a key iron metabolic player influencing cellular metabolic processes and immunogenic functions [1,2,3], thereby controlling tissue homeostasis. Mutations in the *HFE* gene impair the production of the principal iron hormone hepcidin, resulting in iron accumulation in various tissues and the development of hereditary hemochromatosis (HH) [1,4]. While the Hfe actions in hepatocytes are crucial for hepcidin expression and preventing HH [5], it remains unsolved whether *Hfe* expression in other hepatic cells, such as liver sinusoidal endothelial cells (LSECs), hepatic stellate cells (HSCs) and the liver-resident macrophages (Kupffer cells, KCs), may contribute to or modulate iron homeostasis. A recent study by Colluci et al. showed that, despite a high expression in LSECs, Hfe did not contribute to systemic iron homeostasis under basal conditions in adult mice [6]. We demonstrated that Hfe actions in myeloid cells are required to maintain cellular and systemic iron balance in aged *Hfe*^LysMCre^ mice [3]. The latter data raised the question of whether the observed effects of myeloid-*Hfe* stem from the Hfe actions in Kupffer cells or from other non-hepatic macrophages.

Kupffer cells are liver-resident macrophages that originate from the fetal yolk sac [7,8]. Embryonic KCs are self-renewing macrophages that make up the majority of hepatic macrophages [9]. However, in the adult mouse liver, KCs are only minimally replaced by blood monocytes, which rapidly repopulate the adult liver and differentiate into KCs once they are depleted [9]. KCs play a role in responding to a variety of metabolic and inflammatory signals from the blood and from the intra-hepatic environment during homeostasis and disease, such as non-alcoholic fatty liver disease and non-alcoholic steatohepatitis [9]. While KCs are involved in iron scavenging, up until now, the role of *Hfe* in these cells to regulate iron homeostasis cellularly, in the liver and systemically had remained unsolved.

## 2. Results 

**Figure 1:**
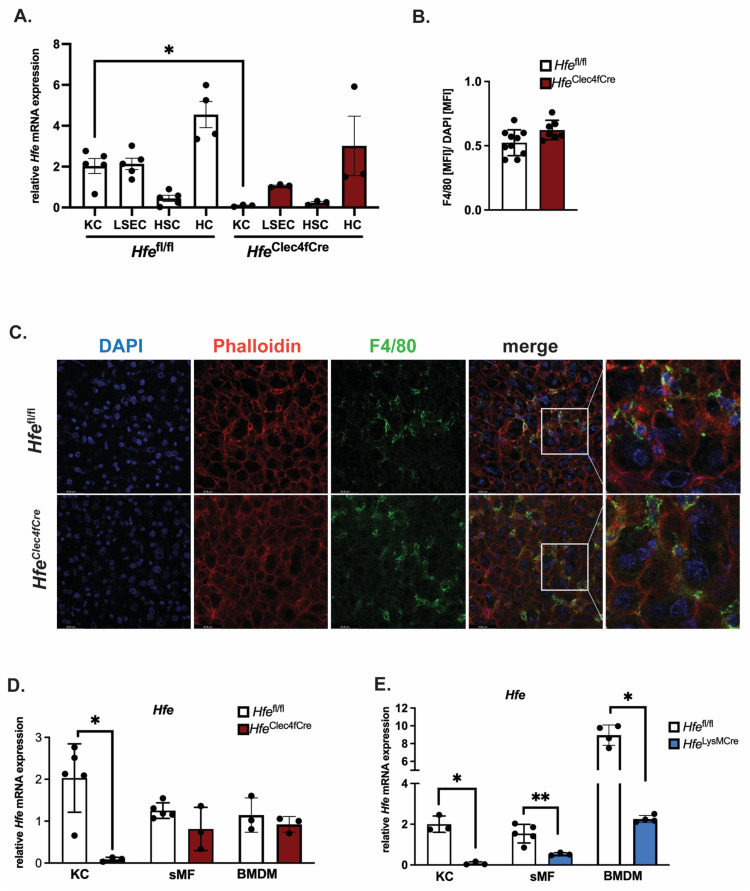
Successful generation of a mouse line specifically lacking *Hfe* in Kupffer cells: *Hfe*^Clec4fCre.^ (**A**) Relative *Hfe*/*Rpl7* mRNA expression of isolated liver cells from *Hfe*^Clec4fCre^ (n = 3 mice) and control mice (n = 3–5); (**B**,**C**) Immunofluorescent staining of F4/80 positive cells in liver tissue (green) with counter staining of DNA with DAPI (blue) and Actin with Phalloidin (red) and quantification of F4/80 positive signals as mean fluorescent intensity (MFI) normalized to MFI of DAPI signals; (**D**,**E**) Relative *Hfe*/*Rpl7* mRNA expression in different macrophage populations of *Hfe*^Clec4fCre^ (n = 3 mice), *Hfe*^LysMCre^ (n = 3 mice) and control mice (n = 3–5): Kupffer cells (KC), splenic macrophages (sMF) and bone-marrow-derived macrophages (BMDMs). Data are representative of one experimental analysis. The experiment has been performed twice with another set of *Hfe*^Clec4fCre^ (n = 3–8 mice), *Hfe*^LysMCre^ (n = 3–5 mice) and control mice (n = 3–5). Significance was tested by using Mann-Whitney-U-test (two-tailed, unpaired, unequal variance.) Significant results were indicated by * *p* < 0.05 or ** *p* < 0.01.

To investigate the role of *Hfe* exclusively in KCs, we crossed *Hfe*^flox^ mice (abbreviated as *Hfe^fl^*^/*fl*^) with mice expressing Cre-T2A-nuclear localization signal-tagged tdTomato (td-Tomato-NLS) in the 3′ UTR of the C-type lectin domain family 4 member F (Clec4f) [10]. The *Hfe*^Clec4fCre^ mutant mice are viable, healthy and do not show physiological abnormalities. The body weight between *Hfe^fl^*^/*fl*^ and *Hfe*^Clec4fCre^ mice is comparable (27.75 g ± 8.7 g vs. 28.95 g ± 3.88 g; n = 3; 9; all females, age: 39–44 weeks). To measure the recombination efficiency of *Hfe* in KCs, we performed a liver perfusion in the *Hfe*^Clec4fCre^ mutant and *Hfe^fl^*^/*fl*^ control mice, followed by differential centrifugation and magnetic cell sorting to obtain Kupffer cells (KC), liver sinusoidal endothelial cells (LSEC), hepatic stellate cells (HSC) and hepatocytes (HC). The purity of these isolated cell types was confirmed by qPCR using cell-specific markers (Figure S1). We show that the *Hfe* expression levels in the KCs and LSECs from the *Hfe^fl^*^/*fl*^ control mice were comparable to each other and were significantly lower than those in the HCs; the lowest expression of *Hfe* was detected in the HSC (Figure 1A). In the *Hfe*^Clec4fCre^ mutant mice, a profound decrease in *Hfe* mRNA expression was detected only in the KCs as it was specific and selective for F4/80 sorted hepatic cells (Figure 1A and Figure S1). Furthermore, the deletion of *Hfe* in the KC did not affect the number of Kupffer cells in the liver of the *Hfe*^Clec4fCre^ mutant mice, demonstrating that *Hfe* does not influence the viability of the Kupffer cell population (Figure 1B,C). In contrast to KCs, the *Clec4f*-Cre mediated excision of *Hfe* did not occur in splenic macrophages (sMF) nor in bone-marrow-derived macrophages (BMDMs) (Figure 1D), reinforcing the finding that *Hfe*^Clec4fCre^ mice carry a selective *Hfe*-deficiency in KCs. This contrasts with previously generated *Hfe*^LysMCre^ mice, where *Hfe* was efficiently recombined in KCs, splenic macrophages and bone-marrow-derived macrophages, being in line with the activity of the *LysM-Cre* promoter, which targets all myeloid-derived cells, including all mature macrophages, such as KC (Figure 1E). 

**Figure 2:**
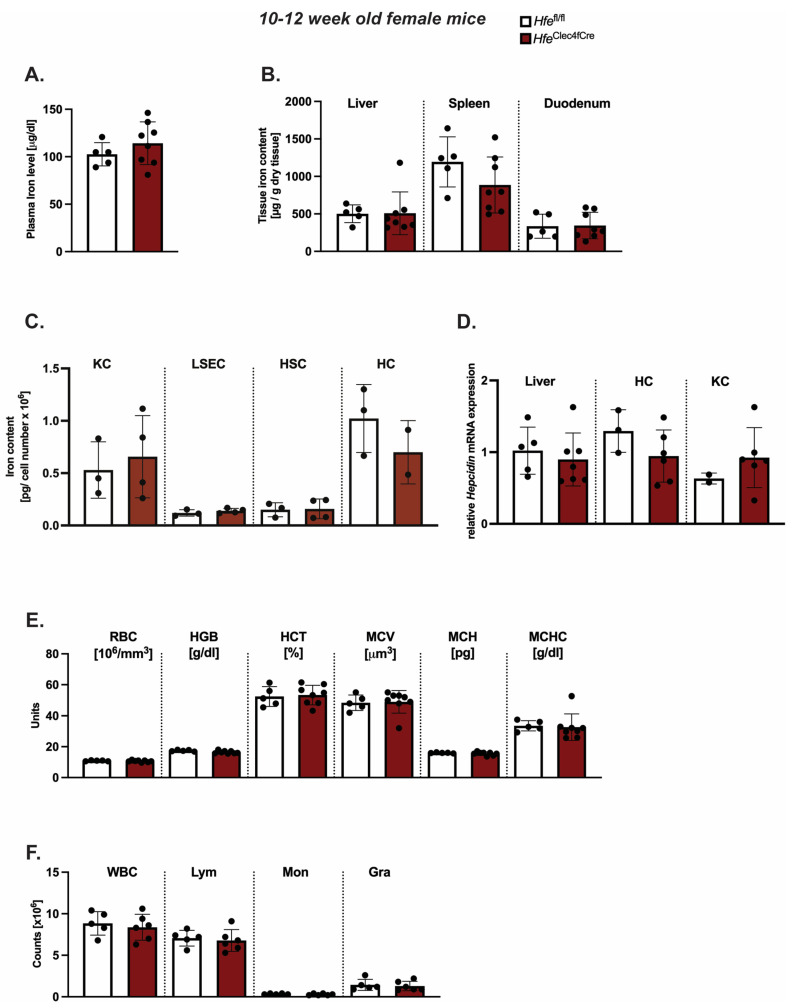
Kupffer cell *Hfe* in young mice is dispensable for cellular and systemic iron homeostasis. (**A**,**B**) Systemic iron levels in *Hfe*^Clec4fCre^ and *Hfe^fl^*^/*fl*^ control mice (n = 8; 5); (**C**) cellular iron levels in isolated primary liver cells from *Hfe*^Clec4fCre^ (n = 4) and *Hfe^fl^*^/*fl*^ control mice (n = 3); (**D**) Relative Hepcidin/*Rpl7* mRNA expression in the liver from *Hfe*^Clec4fCre^ and *Hfe^fl^*^/*fl*^ control mice (n = 8; 5) and isolated primary liver cells obtained from *Hfe*^Clec4fCre^ (n = 6) and control mice (n = 3); (**E**,**F**) Hematological indices in *Hfe*^Clec4fCre^ and *Hfe^fl^*^/*fl*^ control mice (n = 8; 5). Significance was tested by using Mann-Whitney-U-test (two-tailed, unpaired, unequal variance). KC: Kupffer cells; liver sinusoidal endothelial cells (LSECs), hepatic stellate cells (HSCs), hepatocytes (HC).

While *Hfe* actions in myeloid cells contribute to the regulation of macrophages and systemic iron levels in adult mice [3], less is known about the contribution of *Hfe* in Kupffer cells to the regulation of systemic iron levels, hepatic hepcidin expression and hematological indices. To address these questions, we investigated classical iron parameters in 10–12-week-old *Hfe^Clec4fCre^* mice. However, our analysis revealed no significant changes to the iron levels in the blood, liver, spleen or duodenum (Figure 2A,B). We further showed that KCs isolated from the livers of the *Hfe*^Clec4fCre^ mutant and control mice had comparable iron contents, implying that the lack of *Hfe* does not alter the cellular iron phenotype in this cell type (Figure 2C). Similarly, LSEC, HSCs and HCs isolated from the *Hfe*^Clec4fCre^ mice did not show any significant differences in the cellular iron pool compared to the controls (Figure 2C). These data clearly demonstrate that the lack of *Hfe* specifically in KCs does not affect the cellular iron levels, neither in KCs nor in other hepatic cells, emphasizing the fact that the main iron cues in the liver are directed by *Hfe* actions in hepatocytes [5]. In line with the unchanged iron content in the plasma and the liver, we measured no significant difference in the hepcidin mRNA expression in the total liver and in the isolated hepatic and Kupffer cells from the *Hfe*^Clec4fCre^ mice (HCs and KCs) (Figure 2D). 

The same findings were observed when introducing one floxed *Hfe* allele (*Hfe^wt^*^/fl^) with or without the expression of Cre-Recombinase (Figure S2). These data show that Kupffer cell *Hfe* is dispensable for the expression of hepcidin in liver cells and adds to the findings of Lou et al. that Kupffer cells are not required for the iron-mediated expression of hepcidin [11]. As iron metabolism is highly intertwined with the homeostasis of red blood cells, we next investigated hematological indices in the *Hfe*^Clec4fCre^ mice. However, we could not identify any aberrant changes in the red blood cell or hemoglobin concentration (Figure 2E). The counts of white blood cells (WBC), lymphocytes (Lym), monocytes (Mon) and granulocytes (Gra) were unchanged between the *Hfe*^Clec4fCre^ mice and their controls (Figure 2F). Collectively, these findings show that the iron homeostasis is not affected in young *Hfe*^Clec4fCre^ mice and resembles the phenotype of previously published young *Hfe*^LysMCre^ mice [3,5], where *Hfe* is deleted in KCs and in other myeloid populations. 

**Figure 3:**
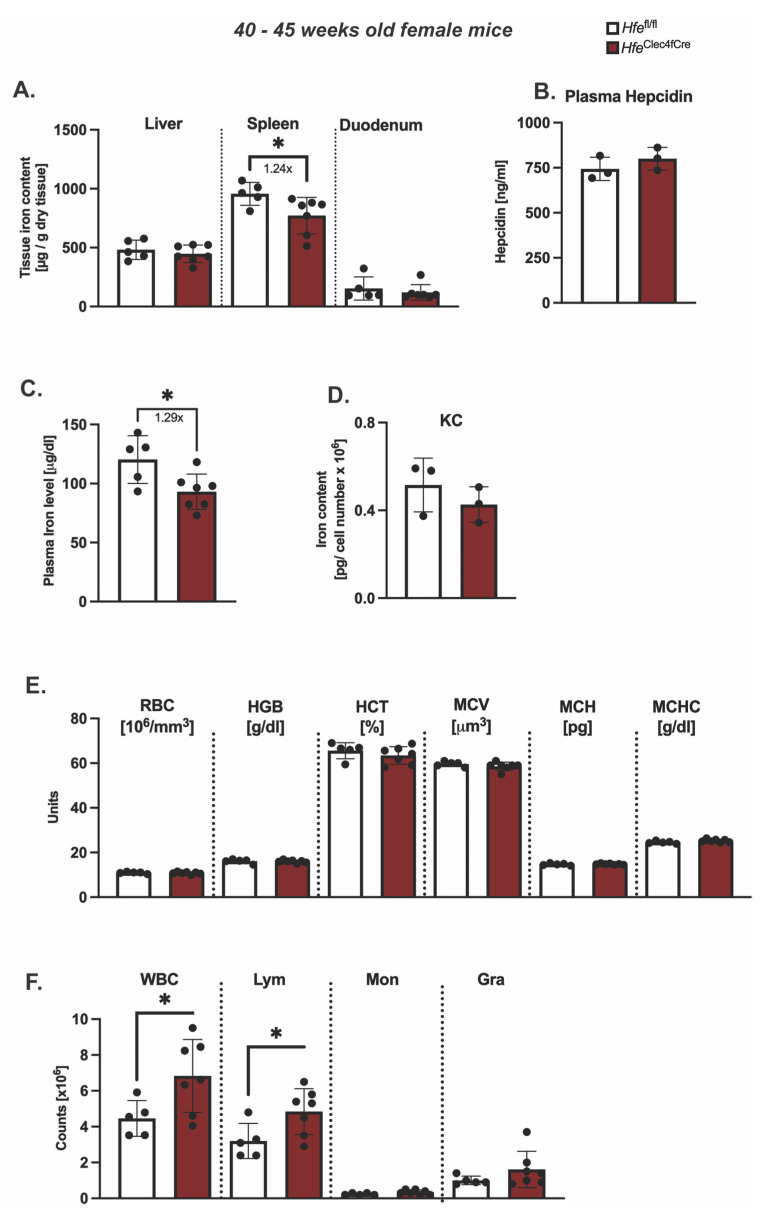
*Hfe* in liver-resident macrophages does not contribute to age-dependent symptomatic manifestation of hemochromatosis. (**A**) Hepatic, splenic, duodenal non-heme iron content in aged *Hfe*^Clec4fCre^ (n = 7) and *Hfe^fl^*^/*fl*^ control mice (n = 5); (**B**) plasma hepcidin levels and (**C**) circulating plasma in aged *Hfe*^Clec4fCre^ (n = 3) and *Hfe^fl^*^/*fl*^ control mice (n = 3); (**D**) Cellular iron content in isolated primary Kupffer cells from *Hfe*^Clec4fCre^ (n = 3) and *Hfe^fl^*^/*fl*^ control mice (n = 3); (**E**,**F**) Hematological indices in *Hfe*^Clec4fCre^ (n = 7) and *Hfe^fl^*^/*fl*^ control mice (n = 5). Data are representative of one experimental analysis. The experiment has been performed twice with another set of *Hfe*^Clec4fCre^ (n = 4 mice) and control mice (n = 3). Significance was tested by using Mann-Whitney-U-test (two-tailed, unpaired, unequal variance). Significant results were indicated by * *p* < 0.05.

However, the symptomatic manifestations of myeloid-*Hfe* deficiency increase with age, as *Hfe*^LysMCre^ mice show mild systemic iron deficiency at 45 weeks old [3]. At present, it is not clear whether the main cellular cues stem from *Hfe* deficiency in KCs or from other myeloid cells. To address this issue, we aged female *Hfe*^Clec4fCre^ mutant mice and their respective controls to 45 weeks of age and analyzed the iron metabolic parameters. We showed that the hepatic iron levels were unchanged in the aged *Hfe*^Clec4fCre^ mice (Figure 3A), and consistent with this, no difference was measured in the serum hepcidin levels when compared to the control mice (Figure 3B). These findings contrast the iron-deficient liver (hepatocytes) phenotype present in the aged *Hfe*^LysMCre^ mice [3]. Of note, a mild (1.2-fold on average) but statistically significant decrease in iron was measured in the spleen and plasma of the aged *Hfe*^Clec4fCre^ mice (Figure 3A,C). The finding that the knock-out of Hfe in KCs lead to an iron decrease in the plasma, which was not the case in the *Hfe*^LysMCre^ mice, is potentially interesting; however, this is currently without any molecular explanation. 

Intriguingly, the total cellular iron levels in the isolated KCs of the *Hfe*^Clec4fCre^ mice were not changed in regard to those in the control cells (Figure 3D). Given that the same iron phenotype was observed in the KC isolated from the aged *Hfe*^LysMCre^ mice [3], our data suggest that Kupffer cells do not change their iron status upon *Hfe* deletion. The comparison between the *Hfe*^Clec4fCre^ and *Hfe*^LysMCre^ mice suggests that a lack of *Hfe* in KCs does not affect the cellular iron levels in liver-resident macrophages and that *Hfe* functions in other myeloid cells contribute to the reported iron-deficient phenotype in *Hfe*^LysMCre^ mice. This hypothesis is underlined by the fact that the knockout of *Hfe* in the myeloid lineage (*Hfe*^LysMCre^) induces a more stringent iron deficiency in multiple organs, such as the liver, spleen and duodenum [3], than *Hfe* deficiency in Kupffer cells.

Lastly, the hematological indices remained largely unaffected in the aged *Hfe*^Clec4fCre^ mice, with the exception of a minimal increase in the number of WBC and Lym (Figure 3E,F). In contrast, the male *Hfe*^Clec4fCre^ mice showed no significant changes in the iron metabolic parameters (Figure S3).

## 3. Discussion

Collectively, our study reports the generation of a new *Hfe* mouse model which lacks the *Hfe* gene specifically in the resident macrophages of the liver. Our findings show that the knock-out of *Hfe* in Kupffer cells, either via Clec4f-Cre- or LysM-Cre-mediated excision, does not alter their cellular iron phenotype, implying that the *Hfe* actions in Kupffer cells are greatly dispensable for cellular iron metabolism. However, *Hfe* is expressed in KCs (Figure 1A), suggesting that KC-*Hfe* may be relevant in processes beyond iron metabolism. Indeed, transcriptomic analysis of tissue-resident macrophages found relative enrichment for the genes involved in lipid metabolism [10,12]. In line with this, Demetz et al. showed that KCs from *Hfe*^-/-^ mice may influence the cholesterol metabolic landscape in the liver, given the expression of lipid genes in KCs and their role in atherosclerosis [13]. The authors demonstrated the KCs’ role in the control of LDL cholesterol homeostasis in mice and patients, a finding that has been corroborated by transcriptomic analysis, revealing the expression of several genes associated with lipid and iron metabolism in KCs [8,13]. 

While our study focuses on iron metabolism, we believe that the function of *Hfe* in KCs may be relevant to any liver disease, including viral infections and liver hepatitis, thus diverting the classical-known role of *Hfe* in iron metabolism to a possibly new role, yet to be established.

## 4. Materials and Methods

### 4.1. Animals Experimentations

*Hfe*^Clec4fCre^ mice were generated by crossing *Hfe^fl^*^/*fl*^ mice [5] with mice expressing Cre-T2A-nuclear localization signal-tagged tdTomato (td-Tomato-NLS) in the 3′ UTR of the C-type lectin domain family 4 member F (Clec4f) [10]. Mice were genotyped for *Hfe* and the presence of *Cre* allele using PCR. Genomic DNA was isolated from 3 mm ear punch using 600 μL Tail Buffer (2M Tris pH 8, 5M NaCl, 0.5M EDTA, 20% SDS, 20 μL Protease K, stock 10 mg/mL). After overnight incubation at 56 °C, 250 μL of 6M NaCl was added, vortexed and centrifuged at 3000 rpm/13 min/RT; 600 μL of the supernatant was mixed with 500 μL Isopropanol, vortexed and centrifuged at 13,000 rpm/7 min; the pellet was washed with 500 μL 70% EtOH, dried for 10 min at room temperature and dissolved in 70 μL water.

*Hfe PCR:* 2 µL of sample DNA was used in 20 mL PCR reaction containing 1× Reaction Buffer supplemented with 1.5 mM MgCl_2_ (Invitrogen, Foster City, CA, USA), 0.25 mM dNTPs (Roth), Platinum Taq Polymerase 0.05 U/mL (Invitrogen) and 0.5 mM of each primer (mHfeExon2Intron2 spanning (F): 5′-CACAGTAAGGGTACGTGGAG-3′, Intron 2 (rev1): 5′-TGGAGACAGTGCAGTAGAGC-3′, Intron 5 (rev2): 5′-AGGGTCACAAACAGCCATAC-3′). The PCR reaction was carried at 94 °C 5 min. One cycle; (94 °C 1 min, 63 °C 1 min, 72 °C 1 min) × 30 cycles; 72 °C 10 min 1 cycle. PCR fragments were resolved on 2% agarose gel (band size: fl/fl: 580 bp, ko/ko: 780 bp, wt/wt: 540 bp).

*Clec4fCre:* 2 μL of sample DNA was used in 20 mL PCR reaction containing One Taq Quick-Load 2× Master Mix (VWR) at working concentration of 1× with 0.5 mM of each primer (rev1: 5′-ACACCGGCCTTATTCCAAG-3′, For: 5′-CAAGAAGTCCACAGGGTGGT-3′, rev2: 5′-GAAAGACCCAAGGGA-3′). The PCR reaction was carried at 94 °C 30 s 1cycle; (94 °C 30 s, 56 °C 30 s, 72 °C 1 min) × 30 cycles; 72 °C 7 min 1 cycle. PCR fragments were resolved on 2% agarose gel (band size: Wildtyp: 313 bp and Transgene: 180 bp).

*Hfe*^Clec4fCre^ mice, on a C57BL/6J genetic background, were used at the ages of 10–12 weeks and 40–45 weeks. All mice were kept under a dark-light cycle (12 h/12 h) with a standard *ad-libitum* diet containing 180 mg/kg iron (Ssniff, Soest, Germany). Mice were euthanized with CO_2_, and blood was withdrawn in a heparin containing tube. For the hematological indices, whole blood samples were evaluated via a Hematology Analyzer (Scil animal care company GmbH, Viemheim, Germany). For plasma analysis, the blood was centrifuged at 5000 rpm for 10 min, the plasma was transferred to a new collecting tube and stored at −80 °C. Other tissues (duodenum, liver, spleen) were harvested, cleaned and stored in −80 °C.

The animal experiments were approved and performed in accordance with the University Animal Care Committee and the Federal Authorities for Animal Research (Regierungspraesidium Tuebingen, Baden-Wuerttemberg, Germany).

### 4.2. Liver Perfusion and Isolation of Liver Cells 

Primary murine hepatocytes (HC), liver sinusoidal endothelial cells (LSEC), Kupffer cells (KC) and hepatic stellate cells (HSC) were isolated from 10-week- and 45-week-old *Hfe*^Clec4fCre^ mutant and control mice through a two-step perfusion method with liver perfusion and liver digest mediums (Life Technologies, Carlsbad, CA, USA) [14]. Thereafter, the liver lobes were mechanically disrupted and passed through 100 μm and 70 μm cell strainers and the cells’ suspension was resuspended in hepatocyte wash medium (Life Technologies). Hepatocytes and non-parenchymal cells (NPC) were separated through centrifugation (50× *g* at 4 °C, for 5 min) and the pellet of hepatocytes was snap frozen. NPC were separated by a gradient centrifugation step with Optiprep (Sigma, Roedermark, Germany) and successively sorted using antibody conjugated with magnetic microbeads against the macrophage (F4/80)- and CD146 (LSEC)-specific marker (Miltenyi Biotec., Bergisch Gladbach, Germany), following the manufacturer’s protocol. 

### 4.3. RNA Isolation and Real-Time PCR

Total RNA was isolated from tissues using Trizol reagent (Invitrogen, Ambion, Foster City, CA, USA) and from primary cells using RNeasy Midi kit (Qiagen, Hilden, Germany) according to the manufacturer’s instruction. On average, 2 μg RNA from the liver and 200 ng of RNA from the cells was used for the reverse transcription reaction. For the real-time PCR, 1 μL of 1:5 diluted cDNA of tissue samples or 1 μL of undiluted cDNA of primary cells was used. 

Data were analyzed using delta, delta Ct (ΔΔCt) method and relative mRNA expression was acquired by normalizing the gene of interest to the reference gene *Rpl7* (data presented in the figures) and *Gapdh.* The following primers were used: *Hfe* (5′-CACCGTCTGTCTGTGCCATCTTCTT-3′ and 5′-ACATAGCCACCCATGGTTCCT-3′), *Albumin* (*Alb*) (5′-AGTGTTGTGCAGAGGCTGAC-3′ and 5′-TTCTCCTTCACACCATCAAGC-3′), *Stabilin-2* (*Stab-2*) (5′-TTTGTGATGAAGGCATGGAA-3′ and 5′-CACAGGCTGTTCCACAGAAA-3′), *F4*/*80* (5′-AGGAGGACTTCTCCAAGCCTA-3′ and 5′-AGGCCTCTCAGACTTCTGCTT-3′), Glial fibrillary acidic protein (*Gfap*) (5′-CGAAGAAAACCGCATCACCAT-3′ and 5′-GGCCTTCTGACACGGATTTG-3′), *Hepcidin* (*Hamp*) (5′-ATACCAATGCAGAAGAGAAGG-3′ and 5′-AACAGATACCACACTGGGAA-3′), *Gapdh* (5′-CCCATTCTCGGCCTTGACTGT-3′ and 5′-GTGGAGATTGTTGCCATCAACGA-3′), *Rpl7* (5′-TCGCAGAGTTGAAGGTGAAGCG-3′ and 5′-CCATCCGAATCTCAGTGCGGTA-3′).

### 4.4. Iron Content Measurement

Plasma iron and non-heme tissue iron in liver, spleen and duodenum measurements were performed as previously described [5]. 

### 4.5. Hepcidin ELISA

Hepcidin was measured in mouse sera using a HAMP ELISA kit (Intrinsic Lifesciences, La Jolla, CA, USA) according to the manufacturer’s instructions. 

### 4.6. Tissue Immunofluorescence Staining

One lobe of liver tissue was snap-frozen in NEG-50 Medium on dry ice and then sliced in 10 μm thick slices using a cryotome. Tissue samples were permeabilized using 0.3% TritonX and blocked in 5% BSA and 0.1% Triton for 30 min. The primary antibody for F4/80 (1:100; 70076S, Cell Signaling, Danvers, MA, USA) was incubated on the liver slices over night at 4 °C. After washing, secondary antibody equipped with Alexa Fluor 488 (1:300) was incubated for 2 h at room temperature. The sections were counterstained with DAPI (1:5000) for 10 min, washed and mounted. Images were taken with a Leica TCS SP8 microscope, using Leica objective HC PL APO 40x/1.30 Oil CS2 506358.

### 4.7. Intracellular Total Iron Content Measurement in Primary Cells

Total iron content in primary cells was measured via total-reflection X-ray fluorescence (TXRF) or inductively coupled plasma optical emission spectrometry (ICP-OES) (Ciros Vision, SPECTRO Analytical Instruments GmbH, Kleve, Germany) after wet-washing the samples with 65% nitric acid (Merck), as previously described [15,16]. In short, cell pellets were digested with high-purity concentrated HNO_3_ (HNO3, 63% AnalR Normapur, VWR International GmbH, Darmstadt, Germany; sub-boiled, DST-1000 Acid Purification System, Savillex, Eden Prairie, MN, USA). After adding 10 μL Gallium solution (100 mg/L in 2% HNO_3_; 1000 mg/L in 2% nitric acid, VWR International GmbH, Darmstadt, Germany), the samples were homogenized through vortexing, and 10 μL of digests in triplicates were dried at 60 °C on pre-cleaned quartz glass carriers. Each sample carrier was analyzed using a S2 Picofox benchtop TXRF with a Mo X-ray tube (50 kV, 600 μA; high-efficiency module, Bruker Nano GmbH, Berlin, Germany) and a live time of 1000 s. A Bayesian deconvolution was applied to the spectra (optimized fit, max. stripping cycles: 100, step width: 1). Iron content is expressed as pg/cell number. 

### 4.8. Data Analysis

The data were analyzed using Prism v.9 (GraphPad) and presented as mean ± SD (standard deviation). For pairwise comparisons, the student *t*-test (two-tailed; unpaired; unequal variance) was used and differences between groups were determined using a log-rank test. * *p* < 0.05, ** *p* < 0.01, are considered statistically significant. 

## Data Availability

Not applicable.

## Supplementary Materials

The supporting information can be downloaded at: https://www.mdpi.com/article/10.3390/ijms24108948/s1.
