# Supplementary material for: Hfe Actions in Kupffer Cells Are Dispensable for Hepatic and Systemic Iron Metabolism
Source: Int J Mol Sci. 2023 May 18;24(10):8948. doi: 10.3390/ijms24108948 (PMC10219340; doi:10.3390/ijms24108948)
Supplement: Supplementary file 1 [file ijms-24-08948-s001.zip › ijms-2358635-supplementary.pdf]

Supplementary Material

Figure S1

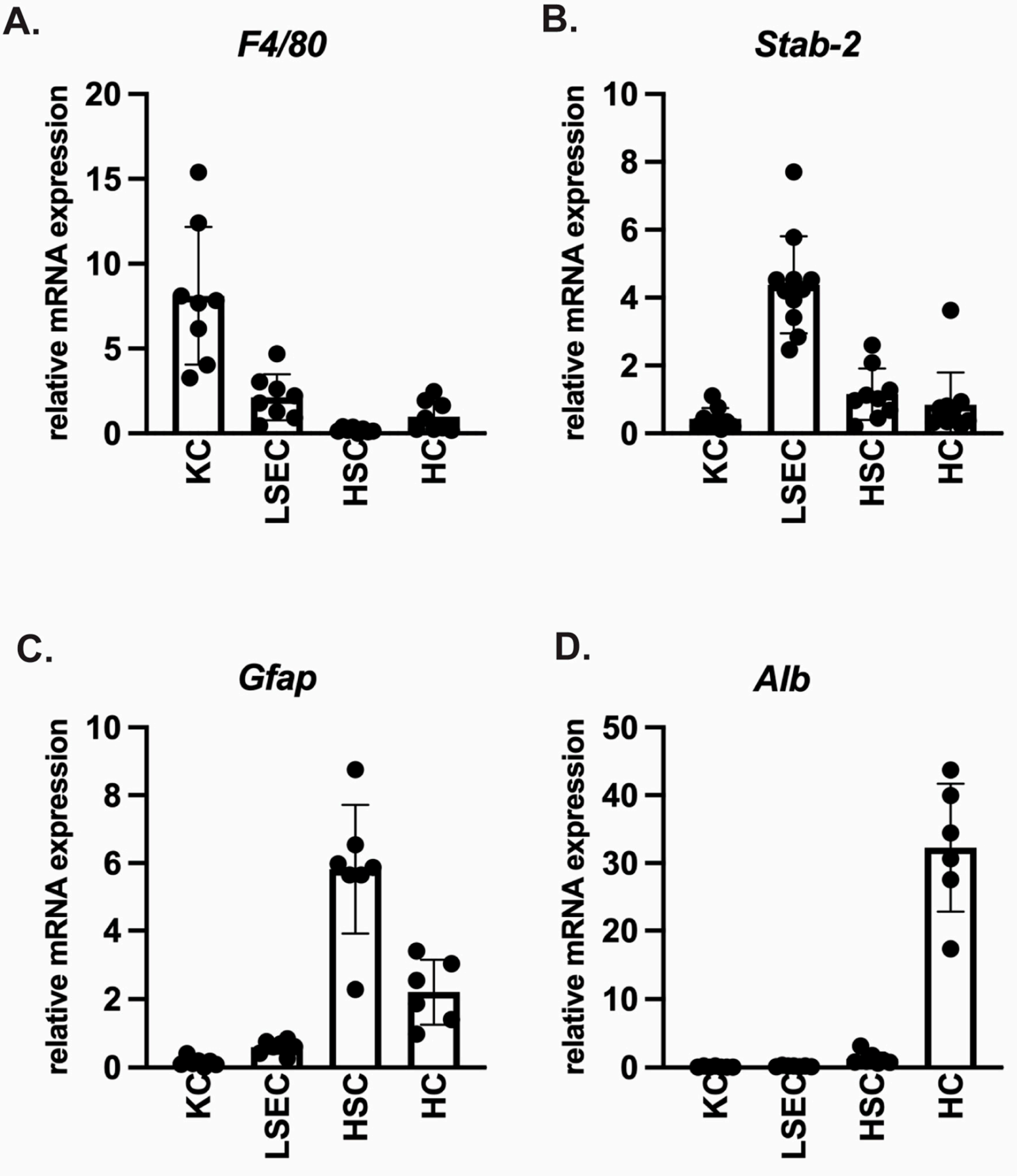

Figure S1: Expression pattern of specific liver cell markers

Expression pattern of specific liver cell markers after liver perfusion and isolation: (A) *F4/80* – Kupffer cells, (B) *Stabilin-2* (*Stab-2*) – liver sinusoidal endothelial cells, (C) *Glial fibrillary acidic protein* (*Gfap*) – hepatic stellate cells and (D) *Albumin (Alb)* – Hepatocytes. Relative mRNA expression of specific liver

cell markers was measured by qPCR and normalized to the expression of *Rpl7* house-keeping gene. Cells were isolated from mixed genotypes (*Hfe*^{flox} and *Hfe*^{Clec4fCre}), n=12 mice.

Figure S2

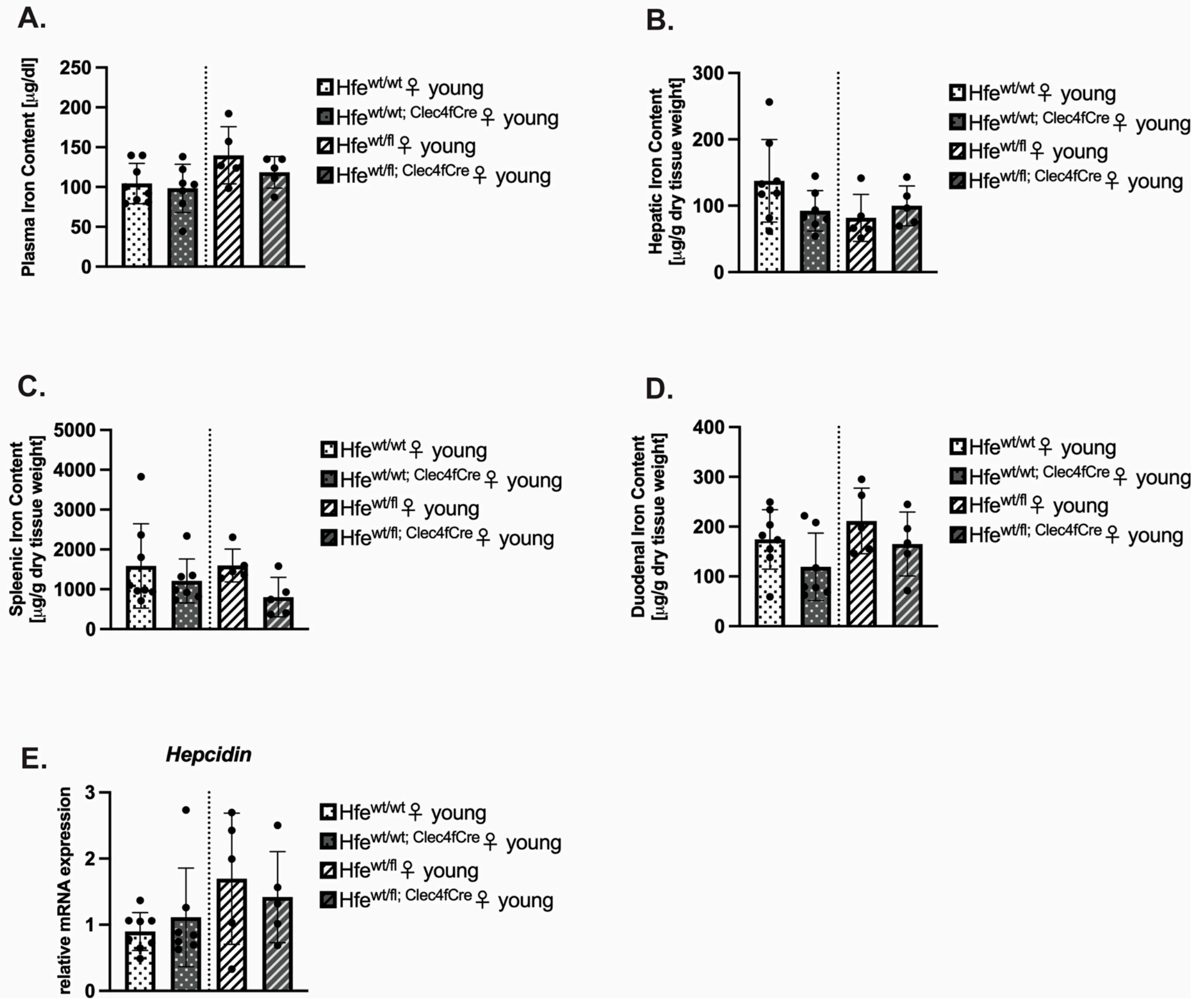

Figure S2: Iron-metabolic parameters in Clec4fCre-mice and in heterozygous mice

(A) Plasma iron level; (B-D) Hepatic, splenic and duodenal non-heme iron content normalized to dry tissue weight; (E) Relative hepatic Hepcidin mRNA expression was measured by qPCR and normalized to the expression of *Rpl7* house-keeping gene. Significance was tested by using Mann-Whitney-U-test (two-tailed, unpaired, unequal variance). Significant results were indicated by * $p < 0.05$ or ** $p < 0.01$

Figure S3

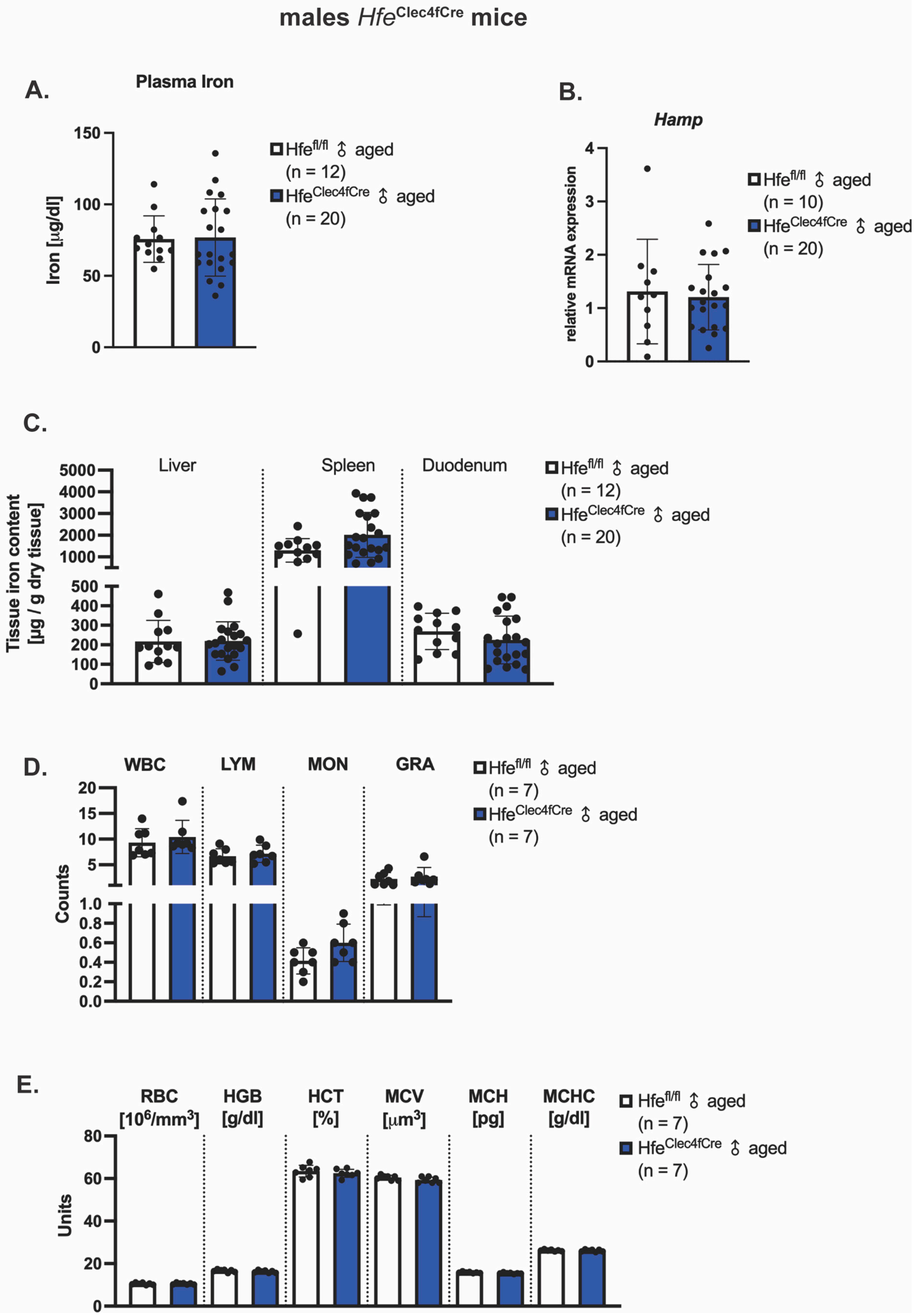
males HfeClec4fCre mice
A.
Plasma Iron
Iron [µg/dl]
150
100
50
0
Hfefl/fl ♂ aged
(n = 12)
HfeClec4fCre ♂ aged
(n = 20)
B.
Hamp
relative mRNA expression
4
3
2
1
0
Hfefl/fl ♂ aged
(n = 10)
HfeClec4fCre ♂ aged
(n = 20)
C.
Liver
Spleen
Duodenum
Tissue iron content
[µg / g dry tissue]
5000
4000
3000
2000
1000
500
400
300
200
100
0
Hfefl/fl ♂ aged
(n = 12)
HfeClec4fCre ♂ aged
(n = 20)
D.
WBC
LYM
MON
GRA
Counts
20
15
10
5
1.0
0.8
0.6
0.4
0.2
0.0
Hfefl/fl ♂ aged
(n = 7)
HfeClec4fCre ♂ aged
(n = 7)
E.
RBC [10^6/mm^3]
HGB [g/dl]
HCT [%]
MCV [µm^3]
MCH [pg]
MCHC [g/dl]
Units
80
60
40
20
0
Hfefl/fl ♂ aged
(n = 7)
HfeClec4fCre ♂ aged
(n = 7)

Figure S3: Iron-metabolic parameters in aged *Hfe*$^{\mathrm{Clec4fCre}}$ male mice

(A) Plasma iron level; (B) Relative hepatic Hepcidin mRNA expression was measured by qPCR and normalized to the expression of *Rpl7* house-keeping gene; (C) Hepatic, splenic and duodenal non-heme iron content normalized to dry tissue weight. Data are representative of *Hfe*$^{\mathrm{Clec4fCre}}$ (n=20) and *Hfe*^{flox} control male mice (n=12). (D-E) Hematological indices in *Hfe*$^{\mathrm{Clec4fCre}}$ (n=7) and *Hfe*^{flox} control mice (n=7). Significance was tested by using Mann-Whitney-U-test (two-tailed, unpaired, unequal variance). Significant results were indicated by * $p < 0.05$ or **$p < 0.01$
